# Climate change and anthropogenic activities shrink the range and dispersal of an endangered primate in Sichuan Province, China

**DOI:** 10.1007/s11356-023-31033-2

**Published:** 2023-11-18

**Authors:** Yunchuan Dai, Dayong Li

**Affiliations:** 1 Key Laboratory of Southwest China Wildlife Resources Conservation (Ministry of Education), China West Normal University, Nanchong, 637009 Sichuan Province China; 2 Institute for Ecology and Environmental Resources, Research Center for Ecological Security and Green Development, Chongqing Academy of Social Sciences, Chongqing, 400020 China

**Keywords:** *Rhinopithecus roxellana*, Climate change, Range shift, Ecological corridor, Sichuan Province

## Abstract

The golden snub-nosed monkey (*Rhinopithecus roxellana*) is a rare and endemic species in China. The population of golden snub-nosed monkeys in Sichuan Province has an isolated genetic status, large population size, and low genetic diversity, making it highly vulnerable to environmental changes. Our study aimed to evaluate the potential impact of climate and land-use changes on the distribution and dispersal paths of the species in Sichuan Province. We used three general circulation models (GCMs), three greenhouse gas emission scenarios, and three land-use change scenarios suitable for China to predict the potential distributions of the golden snub-nosed monkey in the current and 2070s using the MaxEnt model. The dispersal paths were identified by the circuit theory. Our results suggested that the habitats of the golden snub-nosed monkey were reduced under all three GCM scenarios. The suitable habitats for the golden snub-nosed monkey would be reduced by 82.67%, 82.47%, and 75.17% under the RCP2.6, RCP4.5, and RCP8.5 scenarios, respectively, compared to the currently suitable habitat area. Additionally, we found that the density of future dispersal paths of golden snub-nosed monkeys would decrease, and the dispersal resistance would increase. Therefore, relevant wildlife protection agencies should prioritize the climatically suitable distributions and key dispersal paths of golden snub-nosed monkeys to improve their conservation. We identified key areas for habitat preservation and increased habitat connectivity under climate change, which could serve as a reference for future adaptation strategies.

**Supplementary Information:**

The online version contains supplementary material available at 10.1007/s11356-023-31033-2.

## Introduction

The combination of climate change, extreme weather, and pressures from local anthropogenic activities is causing the collapse of global biodiversity and ecosystems (França et al. 2020; Li et al. 2018; Zhang et al. 2019). Climate change and anthropogenic activities can alter the spatial distribution patterns of primates and affect important ecological processes provided by them. A major consequence of future climate change is an increase in extreme weather events that have the potential to alter vegetation structure and species habitats, causing die-offs and increasing extinction risks. Studies exploring the relationship between primate life-history features, climate oscillations, and extreme weather events have shown that primates are extremely sensitive to such disturbances (Wiederholt and Post 2010). Climate change is expected to displace suitable habitats for many species in the future, in which case dispersal capacity is a critical factor in adaptive capacity (Brown and Yoder 2015; Dawson et al. 2011; Dai et al. 2019). However, habitat destruction has reduced landscape permeability and increased resistance to animal movement (Jayadevan et al. 2020), and many primates require wooded habitats, making their dispersal more difficult (Mekonnen et al. 2017). Additionally, spreading in matrix environments transformed by human forces results in threats to survival, such as predation and traffic accidents (LaBarge et al. 2020). If species fail to migrate, their survival will be affected by their living histories and adaptation to the spatial features of their former habitats, such as population size and habitat area (Bellard et al. 2012; Pearson et al. 2014). However, it is concerning that approximately 60% of primate species are currently facing the threat of extinction, and around 75% have declining populations (Estrada et al. 2017). Therefore, synergistic effects between climate change, blocked dispersal paths, and poor adaptive capacity may increase species’ extinction risk.

The golden snub-nosed monkey (*Rhinopithecus roxellana*) is classified as an endangered species on the IUCN Red List due primarily to habitat degradation, fragmentation, and human hunting (Ren et al. 2001). This primate species is predominantly found in mountainous regions at elevations ranging from 1500–3400 m across four provinces in China: Sichuan, Hubei, Shaanxi, and Gansu. The key mountain ranges supporting their population include Qionglai Mountain, Minshan Mountain, Daxiangling Mountain, and Xiaoxiangling Mountain. Presently, the estimated total population of golden snub-nosed monkeys stands at around 22,500, with the majority (approximately 16,000) located in Sichuan and Gansu Provinces, followed by 5500 in Shaanxi Province, and around 1000 in Hubei Province (Arroyo-Rodríguez and Dias 2010; Chang et al. 2012). Displaying arboreal tendencies, they exhibit limited terrestrial locomotion. Active during daylight hours, they demonstrate group-living behavior, forming units ranging from several tens to several hundreds, with smaller family-based subgroups within larger collectives. Their dietary preferences are diverse, consisting primarily of plant-based foods like wild fruits, tender shoots, bamboo shoots, moss, and lichen. Additionally, they consume bark and roots and, in some cases, display a preference for insects, birds, and bird eggs. Mating occurs year-round, with a peak observed during the autumn months (August to October). Gestation lasts approximately 6 months, resulting in offspring typically being born from March to April. Female monkeys typically give birth to a single offspring, although occasional twin births have been recorded. Their lifespan ranges from 20 to 25 years (Qi et al. 2009).

At present, the golden snub-nosed monkey is facing various threats to its survival. Human activities, such as logging, mining, and settlement, have caused habitat loss and reduced their habitat range, making it difficult for them to find food and mates (Huang et al. 2021). In addition, the species has been traditionally hunted for its meat, fur, and other body parts, which are believed to have medicinal properties (Alves et al. 2010). Although hunting is illegal in China, the practice still persists in some remote areas (Ni et al. 2018). Furthermore, the changing climate is affecting the distribution and abundance of the vegetation on which the golden snub-nosed monkey depends (Luo et al. 2015). As temperatures increase and weather patterns change, the vegetation and landscape that these monkeys rely on for food, shelter, and reproduction may also change. As a result, the golden snub-nosed monkey may have to adapt to these changes in order to survive. This could involve changes in behavior, such as altering their diet or range, or physiological changes, such as adapting to new temperature or moisture conditions. However, if the changes are too severe or occur too quickly, the golden snub-nosed monkey may not be able to adapt quickly enough, and their populations could decline or even go extinct. Hence, identifying and protecting suitable dispersal routes can help ensure the long-term survival of the golden snub-nosed monkey and other vulnerable species.

Presently, habitat conservation and the prediction of suitable distributions have gained significant prominence within the realm of primate conservation (Flesher 2015; Ren et al. 2017). These research findings provide a solid scientific basis for conservation efforts, whether in the natural habitat (in situ) or in controlled environments (ex situ) for primates. While a range of research methods exists for habitat conservation and prediction, the MaxEnt model and Circuitscape theory hold notable favoritism for simulating primate habitats and corridors (Zhao et al. 2019). The MaxEnt model is a widely embraced tool in ecological research, employed to forecast species distribution and assess habitat suitability (Phillips and Dudík 2008). Leveraging presence-only data, environmental variables, and a sophisticated machine learning algorithm, it estimates the likelihood of species occurrence across a given landscape. Notably, MaxEnt excels in handling incomplete data and generating reliable predictions even in cases of limited information availability. In the realm of primate habitat simulation, MaxEnt proves invaluable by effectively modeling the environmental conditions conducive to primate presence (Phillips and Dudík 2008). Circuitscape stands as a spatially explicit software application integral to landscape ecology. Its primary function lies in modeling landscape connectivity and conducting in-depth analyses of connectivity patterns. It accomplishes this by simulating the flow of movement and gene dispersal across landscapes, with a particular focus on identifying potential wildlife corridors or pathways. Circuitscape also factors in resistance values associated with various landscape features, a key aspect in pinpointing critical areas for sustaining connectivity between habitat patches (McRae and Beier 2007; Walpole et al. 2012). In the context of primate corridor simulation, Circuitscape plays a pivotal role in evaluating landscape connectivity and identifying indispensable corridors that facilitate primate movement, gene flow, and population viability. The combined utilization of MaxEnt and Circuitscape empowers researchers not only to forecast suitable primate habitats but also to comprehensively comprehend and strategize for the effective design of corridors (Zhao et al. 2019). This integrated approach proves instrumental in the conservation and management of primate populations.

Over the past two decades, the Chinese government has made significant efforts to raise awareness about wildlife protection. Since 1989, Sichuan Province has implemented various crucial projects, including the construction of the Yangtse River shelter-forest system, the protection and development of natural forest resources, the return of cultivated land to forests and grasses, the creation of nature reserves, and the building of forest urban parks. As a result, the survival rate of the golden snub-nosed monkey has seen a significant improvement, leading to substantial population growth. For instance, in Shennongjia National Park of China, the golden snub-nosed monkey population has surged from 1218 individuals in 2005 to well over 1400 individuals by 2023. Furthermore, the habitat area has expanded from 210 km^2^ to its current expanse of 354 km^2^. However, in some areas of Sichuan Province, the distribution range of the golden snub-nosed monkey is still decreasing (Dong et al. 2019). The golden snub-nosed monkey is a highly sensitive species and a valuable indicator of climate change (Luo et al. 2012). Monitoring their habitats and populations can provide essential insights into the effects of climate change on forest ecosystems. By studying the impact of climate change on this species, we can gain a better understanding of the complex interactions between climate change and biodiversity. This understanding can help us identify conservation strategies and management plans to mitigate the effects of climate change on the species. Moreover, the golden snub-nosed monkey is a critical component of China’s temperate forests’ biodiversity, which is home to many other species of plants and animals. This information can be used to develop conservation strategies to protect the overall biodiversity of these ecosystems. To develop a comprehensive conservation action plan for the golden snub-nosed monkey, it is crucial to conduct a habitat suitability and dispersal path assessment. Our results can provide valuable insights for situ and ex situ conservation planning of the golden snub-nosed monkey.

## Material and methods

### Study area and species occurrence

**Fig. 1:**
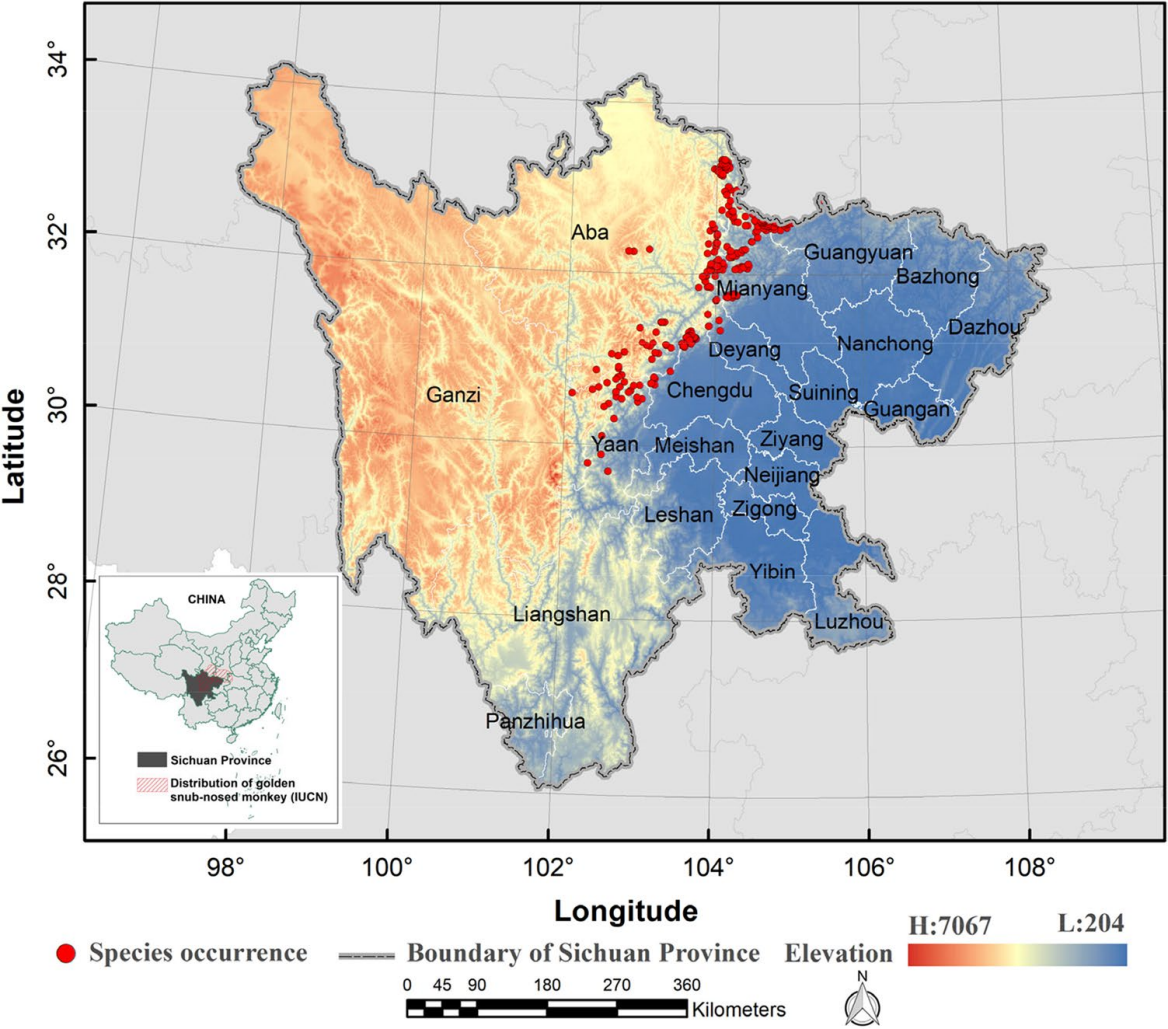
Location of Sichuan Province, China

Sichuan Province is located in the southwestern hinterland of China, at the upper reaches of the Yangtze River. It spans between longitude 97°21′ ~ 108°12′ E and latitude 26°03′ ~ 34°19′ N and covers an area of 486,000 km^2^, ranking fifth in China (Fig. 1). The province boasts a high diversity of terrestrial vertebrates, making it one of the regions with the most biodiversity in the southwest of China. It is situated in the transition zone between the Tibetan Plateau and the Middle-Lower Yangtze plains. With its abundant animal and plant resources, Sichuan Province is a valuable biological gene bank in the world (López-Pujol and Zhao 2004). Since 2018, the Sichuan Forestry Bureau, acting as the leading management and protection agency of biological resources, has established 129 nature reserves, covering a total area of 7,346,100 km^2^. These reserves account for 88.51% of the total area of nature reserves in the province. Within these reserves, the golden snub-nosed monkey is distributed across 29 nature reserves.

We utilized a comprehensive approach to investigate the distribution of golden snub-nosed monkeys. To determine the field survey areas, we analyzed the historical distribution information of the monkey population, taking into account factors such as local vegetation types, elevation, and accessibility. Once we had established our survey areas, we utilized the transect method to record the spatial distribution of the monkeys. We walked along the transects, recording the location of all observed individuals of golden snub-nosed monkeys. We then utilized a GPS device to record the precise location of each sighting. To avoid spatial autocorrelation of occurrences affecting the model results, we set up a 1 km^2^ grid based on the average daily movement distance (approximately 1 km) of the golden snub-nosed monkey (Li et al. 2005), kept one random occurrence in the kilometer grid of golden snub-nosed monkey, and eventually gained 387 valid occurrences (Fig. 1; Appendix 1).

### Data collection

The 19 bioclimatic factors (bio1-bio19; Appendix 2) under current and future climatic conditions were extracted from the WorldClim database. The comprehensive details of the analyzed data are presented in Table 1. Since the study could not obtain the HII and DEM data in the 2070s, we kept these two variables stationary in the prediction model (Stanton et al. 2012).

**Table 1:** Data information

Data	Category	Sources	Spatial resolution
Current climate data (1950–2000)	/	https://www.worldclim.org/	1 km
Future climate data (RCP2.6, RCP4.5, RCP8.5; 2061–2080)	BCC-CSM1-1	https://www.worldclim.org/	1 km
	CCSM4	https://www.worldclim.org/	1 km
	HadGEM2-AO	https://www.worldclim.org/	1 km
Current land cover	/	http://www.globallandcover.com/	30 m
Future land cover	/	http://data.ess.tsinghua.edu.cn/data/Simulation/	30 m
Human Influence Index	/	http://sedac.ciesin.columbia.edu/	1 km
DEM	/	http://www.gscloud.cn/	30 m

### Variables processing

We resampled all variables to 1 km spatial resolution in a uniform coordinate system. Multicollinearity of variables was excluded by eliminating relational variables where Pearson’s |*r*|> 0.8 (Appendix 3) (Cord et al. 2014; Dai 2022; Searcy and Shaffer 2016). Finally, eight variables remained to compute the model and to project the golden snub-nosed monkey’s current and future habitat (Li et al. 2018). The remained variables included ELE, HII, LUCC, Bio4, Bio7, Bio12, Bio13, and Bio15.

### Species distribution model and dispersal analyses

MaxEnt model was used to project the habitat distribution for the golden snub-nosed monkey. This approach is considered one of the best-performing algorithms in predicting species distribution (Phillips and Dudík 2008). The model’s operational parameters were informed by insights from prior research (Dai et al. 2019; Li et al. 2019; Ramirez-Villegas et al. 2014). The area under the receiver operating characteristic (ROC) curve, known as the AUC, was used to measure model performance. The value of AUC ranges from 0 to 1, with a value of 1 signifying perfect model performance (Phillips et al. 2006). Using the average logistic threshold value of maximum training sensitivity plus specificity (MTSPS) output by MaxEnt (Dai et al. 2019), we divided the cells into two: suitable and unsuitable. Any cells with a value greater than the MTSPS threshold indicate a suitable cell for the monkeys.

To ensure that the suitable habitats were realistic, we applied filters based on the monkey’s minimum home range size and daily path length. Specifically, we removed patches with an area less than 7.4 km^2^ and a distance greater than 2.1 km from the nearest patch (Tan et al. 2007). Circuitscape was applied to identify the potential dispersal path of golden snub-nosed monkeys between current and future suitable habitats. Circuitscape uses circuit theory to simulate connectivity in heterogeneous landscapes. Landscape is represented as a conductive surface, with low resistances assigned to landscape feature types that are most permeable to movement or best promote gene flow and high resistances assigned to movement barriers (McRae and Beier 2007; Walpole et al. 2012).

## Results

### Model performance

**Fig. 2:**
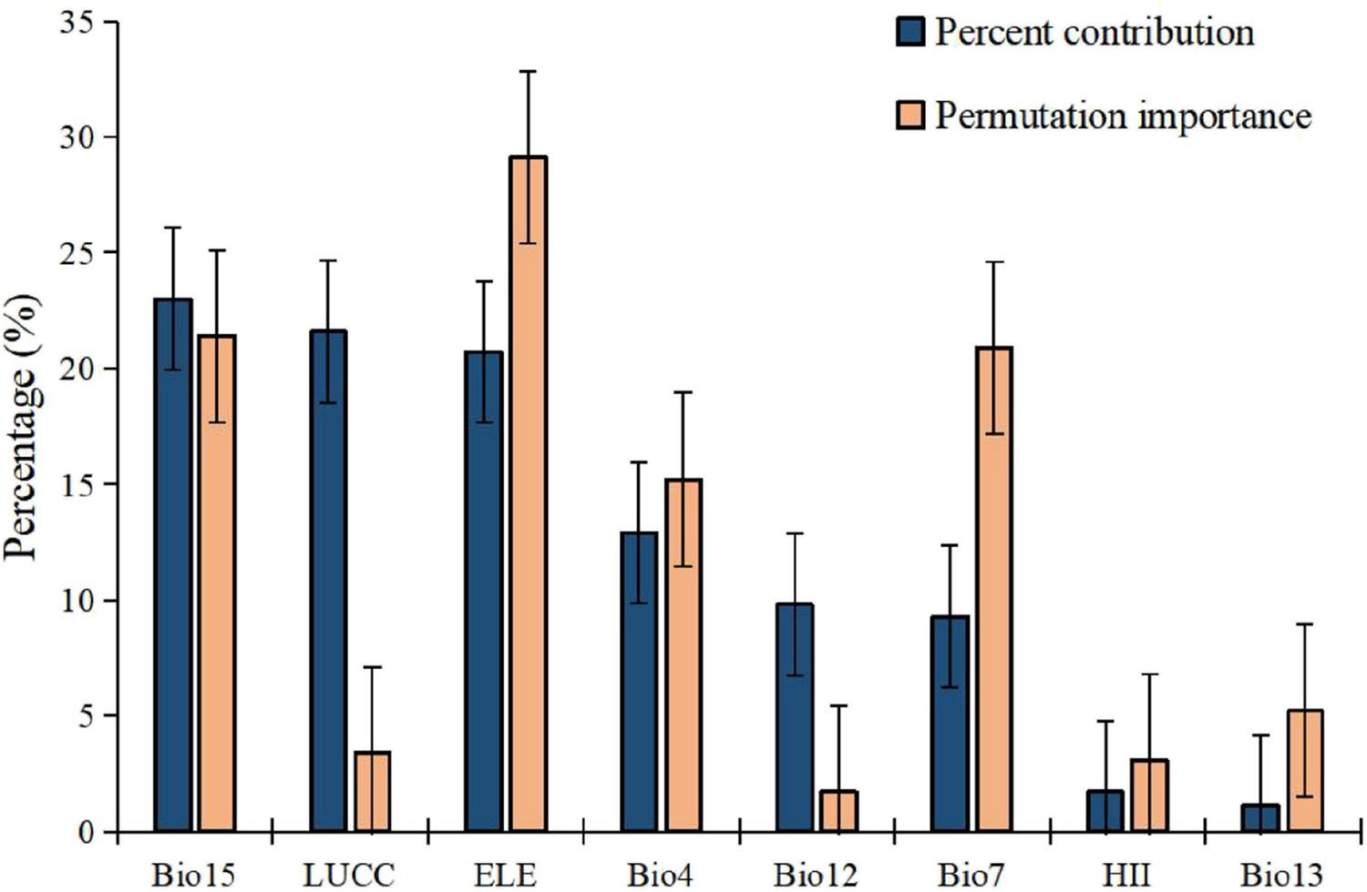
The estimates of relative contributions of the environmental variables to the MaxEnt model

Generally, climatic variables, land cover, and elevation contributed significantly, with Bio15, LUCC, and ELE providing 23%, 21.6%, and 20.7% of the distribution of the golden snub-nosed monkey, respectively (Fig. 2). The AUC of the model was 0.968, which showed a high accuracy of the model results (Appendix 4).

### Projected distributions of the golden snub-nosed monkey

**Fig. 3:**
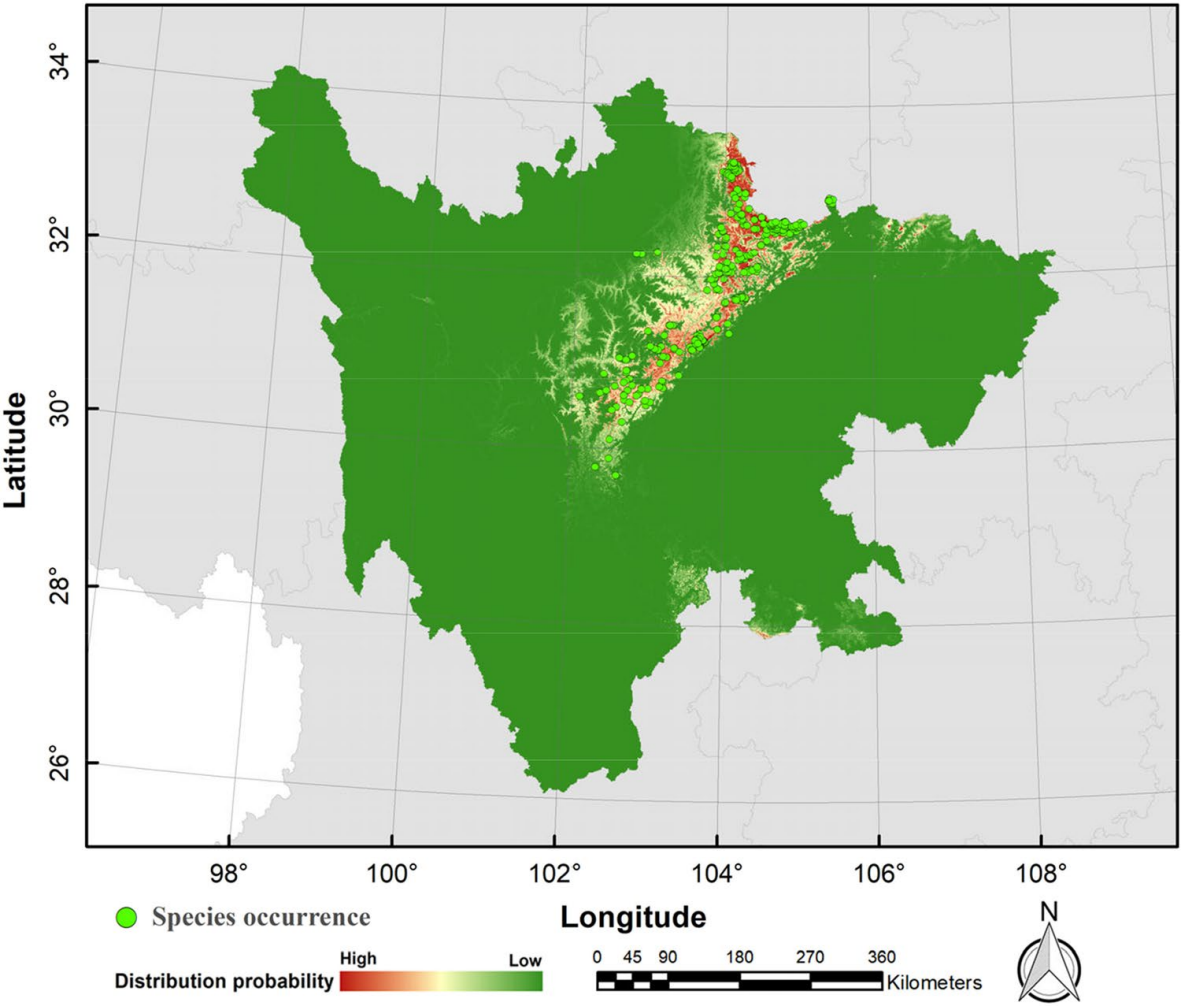
The current distribution probability of golden snub-nosed monkey (*Rhinopithecus roxellana*) in Sichuan Province, China

**Fig. 4:**
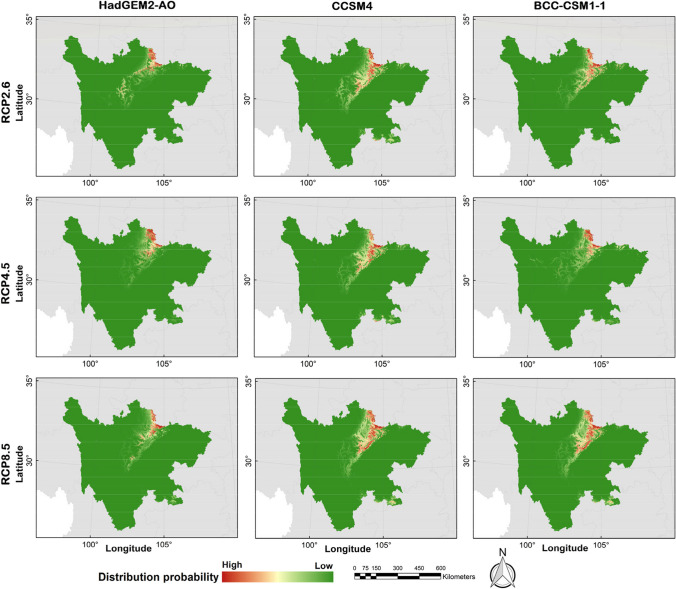
The future distribution probability of golden snub-nosed monkey (*Rhinopithecus roxellana*) in Sichuan Province, China

**Fig. 5:**
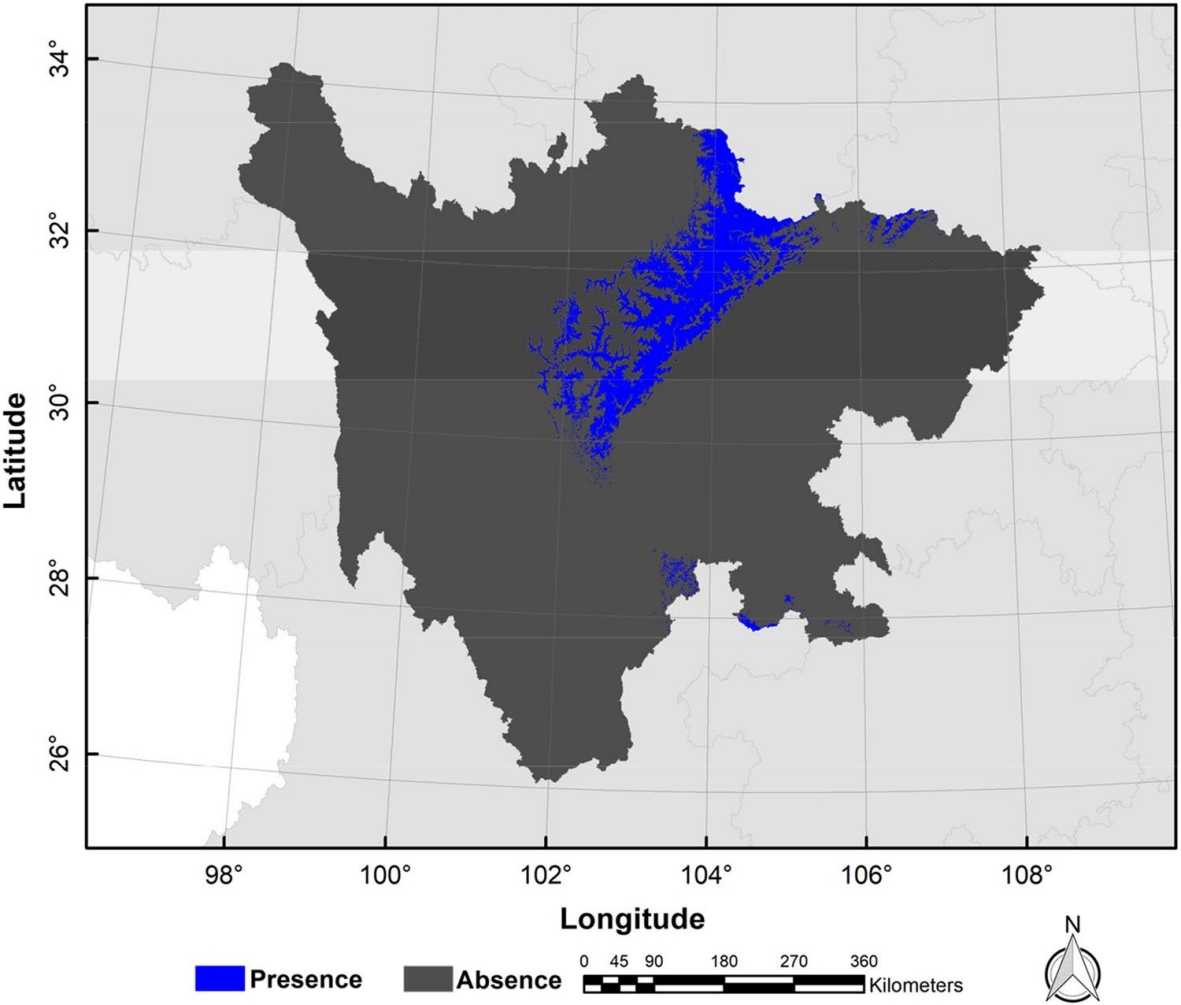
The current binary distribution map of golden snub-nosed monkey (*Rhinopithecus roxellana*) in Sichuan Province, China

**Fig. 6:**
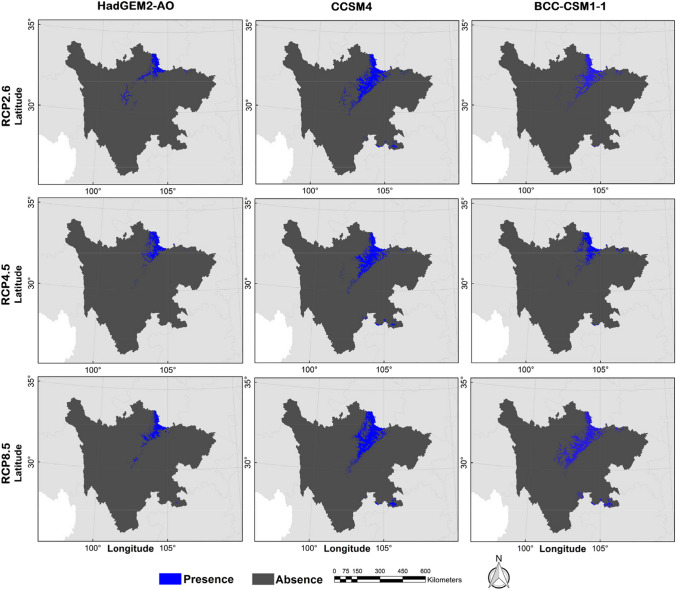
The future binary distribution maps of golden snub-nosed monkey (*Rhinopithecus roxellana*) in Sichuan Province, China

**Fig. 7:**
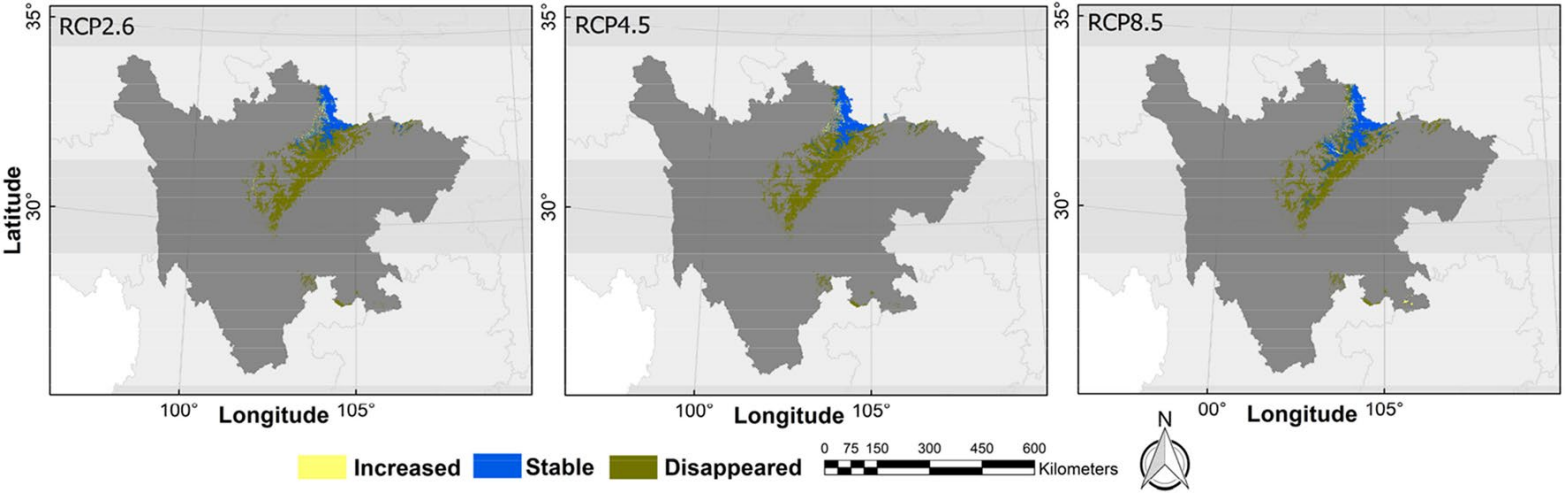
Spatial variation of the habitat for golden snub-nosed monkey (*Rhinopithecus roxellana*) in Sichuan Province, China. Future habitats are the intersection area of the three GCMs (HadGEM2-AO, CCSM4, and BCC-CSM1-1)

The possible distribution probabilities of the golden snub-nosed monkey are shown in Figs. 3 and 4, and the binary distribution maps are shown in Figs. 5 and 6 (MTSPS = 0.1535). In the current climate scenario, the suitable habitat area for golden snub-nosed monkeys was 31,592.64 km^2^, mainly distributed in 12 administrative regions of Sichuan Province (Table 2). By the 2070s, the areas of suitable habitat were predicted to be reduced (Fig. 7). Under the scenarios of RCP2.6, RCP4.5, and RCP8.5, the suitable habitats were 5476.38 km^2^, 5538.41 km^2^, and 7844.39 km^2^, respectively, which were 82.67%, 82.47%, and 75.17% less than the current habitat areas. The future habitats were mainly located in Aba, Mianyang, and Guangyuan in northern Sichuan Province, with a few suitable habitats in Bazhong, Luzhou, and Yaan (Fig. 6).

**Table 2:** The suitable distribution area of the golden snub-nosed monkey

Location	Current (km^2^; %)		RCP2.6 (km^2^; %)		RCP4.5 (km^2^; %)		RCP8.5 (km^2^; %)	
	Area	Proportion	Area	Proportion	Area	Proportion	Area	Proportion
Aba	15,339.26	48.55	3128.42	57.13	2833.98	51.17	3837.20	48.92
Bazhong	215.71	0.68	7.89	0.14	0.98	0.02	0.00	0.00
Chengdu	1038.37	3.29	0.00	0.00	0.00	0.00	2.02	0.03
Deyang	576.60	1.83	0.00	0.00	0.00	0.00	0.00	0.00
Ganzi	1515.71	4.80	21.86	0.40	0.00	0.00	0.00	0.00
Guangyuan	1608.43	5.09	289.98	5.30	292.95	5.29	477.53	6.09
Leshan	94.24	0.30	0.00	0.00	0.00	0.00	0.00	0.00
Liagnshan	298.32	0.94	0.00	0.00	0.00	0.00	0.00	0.00
Luzhou	44.69	0.14	0.00	0.00	0.00	0.00	52.62	0.67
Mianyang	6858.23	21.71	2027.39	37.02	2410.46	43.52	3435.18	43.79
Yaan	3604.49	11.41	0.84	0.02	0.04	0.00	39.84	0.51
Yibin	398.59	1.26	0.00	0.00	0.00	0.00	0.00	0.00

### Dispersal paths of the golden snub-nosed monkey

**Fig. 8:**
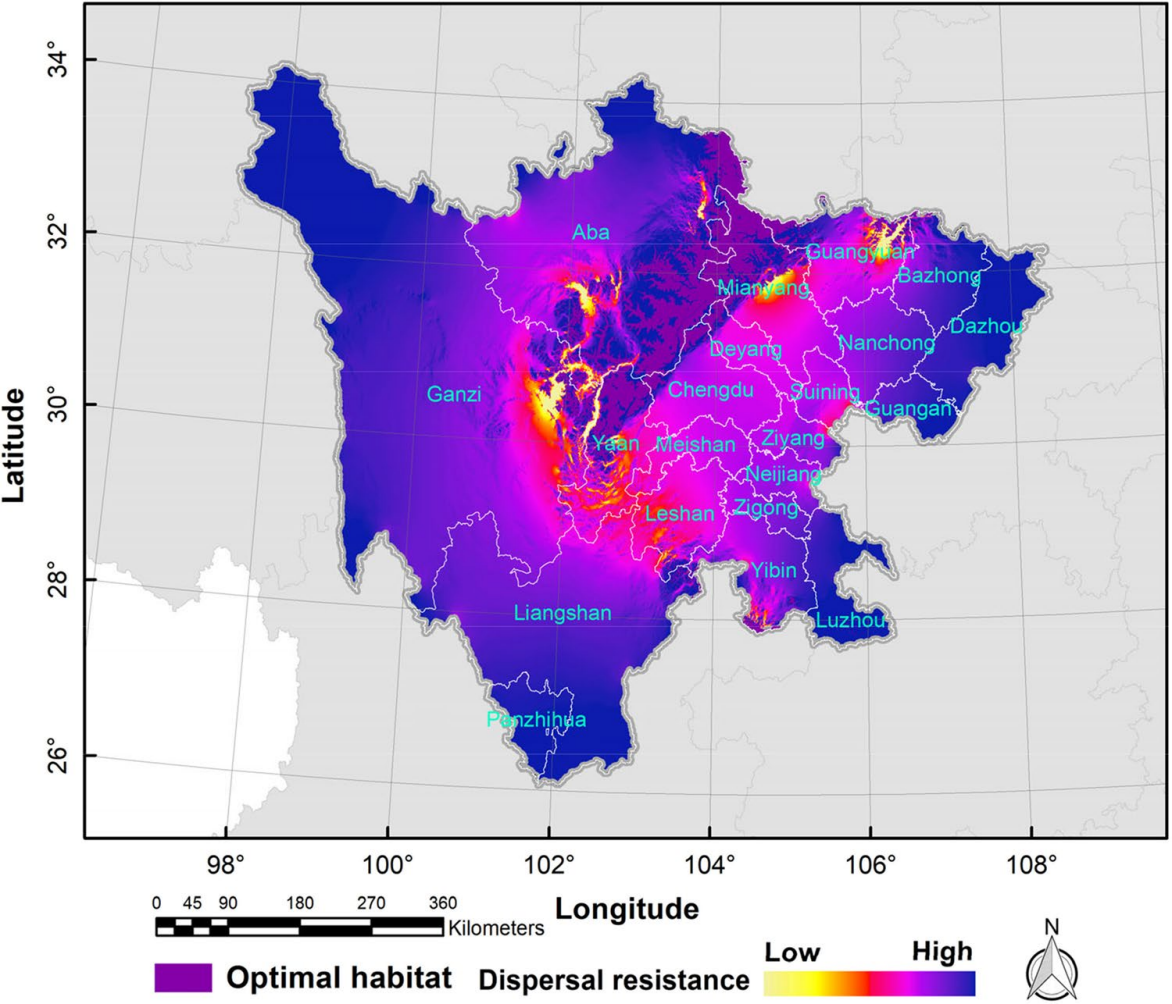
The current potential dispersal paths for golden snub-nosed monkey (*Rhinopithecus roxellana*) in Sichuan Province, China

**Fig. 9:**
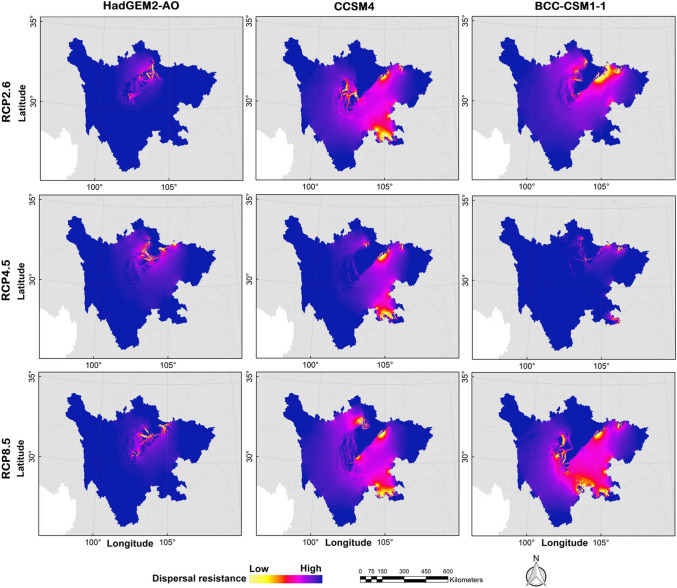
The future potential dispersal paths for golden snub-nosed monkey (*Rhinopithecus roxellana*) in Sichuan Province, China

Under the current climate conditions, the current values in northern Guangyuan, central Mianyang, western Leshan, southern Yaan, eastern Ganzi, central Aba, southern Leshan, and southern Yibin were relatively higher, with less spreading resistance for golden snub-nosed monkeys. The dispersal paths roughly showed a “C”-shaped direction (Fig. 8). By the 2070s, the dispersal paths of golden snub-nosed monkeys under different climate conditions showed an increase in spreading resistance and a decrease in density. In the 2070s, there were some differences of dispersal paths in spatial distributions (Fig. 9). However, the dispersal paths all showed an increase in spreading resistance and a decrease in density compared to the current scenario.

Based on the GCM of HadGEM2-AO, the dispersal paths of golden snub-nosed monkeys under the RCP2.6, RCP4.5, and RCP8.5 scenarios showed the following features: the current values in northern Ya’an, southeastern Aba, northwestern Mianyang, and northwestern Guangyuan were relatively higher, whereas the diffusion resistance of golden snub-nosed monkeys was lower, and the dispersal paths were in a southwest-northeast direction with less resistance. Based on the GCM of CCSM4, the dispersal paths of golden snub-nosed monkeys under the RCP2.6, RCP4.5, and RCP8.5 scenarios showed the following features: the current values in eastern Aba, central Mianyang, northern Guangyuan, southeastern Yibin, and southern Luzhou were relatively higher, and the dispersal paths of golden snub-nosed monkeys were approximately in the north–south direction with less resistance. Based on the GCM of BCC-CSM1-1, the dispersal paths of golden snub-nosed monkeys under the RCP2.6, RCP4.5, and RCP8.5 scenarios showed the following features: the current values in southern Aba, eastern Ganzi, central Mianyang, northern Guangyuan, and southern Luzhou were relatively higher, and the dispersal paths of golden snub-nosed monkeys were in a “V”-type direction with less resistance (Fig. 9).

## Discussion

Recent increases in temperature and rainfall may have expanded habitat connectivity for some populations, while for primate populations, forest fragmentation is expected to intensify, reducing habitat quality and leading to reduced home ranges (Xiao et al. 2003). Surviving in a fragmented and limited area under the influence of geographic isolation, golden snub-nosed monkeys have disappeared from the south and northeast of Sichuan, and the current population of the species is distributed only in an isolated area (Li et al. 2002). Climate change and anthropogenic activities are the two major factors driving these changes in the distribution of golden snub-nosed monkeys (Luo et al. 2015). The two factors are likely to be the main threats to the degradation and fragmentation of the habitats for golden snub-nosed monkeys. Therefore, the habitat and dispersal paths assessment of golden snub-nosed monkeys under climate change can help reduce the negative impact of climate change on this species. Predict the direction of habitat succession, formulate protection countermeasures to adapt to climate change in advance, and store the necessary living space for golden snub-nosed monkeys to cope with future climate change.

Our findings indicate that Bio15 (precipitation seasonality) and ELE significantly impact the distribution of golden snub-nosed monkeys. Previous studies proved that Bio15 plays a crucial role in shaping wildlife habitat suitability (Li et al. 2018; Zhang et al. 2019). Since the golden snub-nosed monkey relies on plant material for food, the availability of water is critical to its survival. Changes in precipitation seasonality patterns, such as shifts in timing, intensity, or frequency, can affect the availability of food and water resources, ultimately affecting the golden snub-nosed monkey’s distribution (Zhang et al. 2019). To further research and conservation efforts, it would be beneficial to investigate how climate change and other environmental factors could impact precipitation seasonality patterns and, in turn, affect the distribution of the golden snub-nosed monkey. Elevation can also influence temperature, precipitation, and vegetation patterns, which are all critical factors in determining the distribution of the golden snub-nosed monkey (Li et al. 2018). Future research and conservation efforts should aim to investigate how changes in elevation due to climate change could impact the golden snub-nosed monkey’s habitat suitability and how targeted conservation interventions can address these changes.

The impact of carbon dioxide emissions on the future distribution of wildlife habitats varies significantly depending on the specific RCP scenarios being considered (Dai et al. 2021; Tang et al. 2022). We have unveiled a fascinating finding that the projected habitat degradation of the golden snub-nosed monkey is likely to be less severe under RCP8.5 scenarios than under RCP2.6 and RCP4.5 scenarios. Our study raises intriguing questions about the underlying mechanisms driving this trend. One possible explanation is that increased carbon dioxide levels may stimulate plant growth in the southwest mountains of China, thereby enhancing food resources for the golden snub-nosed monkey. This could potentially counterbalance the negative effects of habitat degradation triggered by climate change. Alternatively, this phenomenon may stem from intricate ecological interactions within the golden snub-nosed monkey’s ecosystem. Changes in predator or prey behavior, abundance, or distribution, for instance, could yield unforeseen impacts on this species’ habitat distribution. Further research is necessary to comprehensively understand these complex interactions and their effects on biodiversity. Our study highlights the importance of considering multiple RCP scenarios when anticipating the impact of climate change on species distribution. Understanding the mechanisms behind these trends is essential for developing effective conservation strategies aimed at safeguarding endangered species.

Anthropogenic activities have caused changes in the distribution areas of mammals, leading to a reduction in their original range and affecting changes in their suitable habitat and preferred climate (McGuire et al. 2016; Pineda-Munoz et al. 2021). Mammals surviving in environments disturbed by humans show reduced activity, and it appears as if wildlife associates human activity with the risk of mortality (Frid and Dill 2002). There is a significant overlap between the altitudinal range of anthropogenic activities and the distribution of suitable habitats for golden snub-nosed monkeys, indicating a potentially competitive relationship between human habitation and these primates (Zhao et al. 2019). Golden snub-nosed monkeys are typically found in warmer, wetter, and lower altitude areas. However, due to agricultural development and increased human population in these areas, golden snub-nosed monkeys have gradually retreated to higher altitude areas that are more difficult for humans to access (Nüchel et al. 2018). Our findings suggest that LUCC has a significant impact on the distribution of golden snub-nosed monkeys. However, we loaded data for all land-use types at the early stage of modeling instead of analyzing each land-use type separately. Consequently, determining the contribution rate of a particular land-use type to the distribution of this species is challenging. Nevertheless, it is evident that forests are crucial for the current and future distribution of golden snub-nosed monkeys. This is because both current and future LUCC data include forests, and the species is primarily a forest-dwelling species. Therefore, it is crucial to strengthen forest protection and reduce human activities’ interference to ensure the long-term survival of this species.

Although there has been more researches on the habitat evaluation of golden snub-nosed monkeys, most of the research in the past was on a single protected area. The previous studies predicted that the suitable habitat area for golden snub-nosed monkeys in Yunnan Province of China would be reduced by 15% in 2050 (Li et al. 2011), and the potential habitat area for golden snub-nosed monkeys in Vietnam would be reduced by 20% compared to the current habitat area (Van Manh et al. 2010). Our findings were similar to the previous studies which predicted that the Shennongjia forest area of China would lose 70% of the suitable habitat for golden snub-nosed monkeys by 2050 (Luo et al. 2015). It is worth noting that, due to the selection of diverse regions, varying scales, different climate scenarios, and a range of variables, the model results may exhibit some inherent biases (Yu et al. 2022; Luo et al. 2015). The reported records of the genus golden snub-nosed monkey species indicated that the species was most severely affected by climate change and was predicted to increase its fragility. We took the whole of Sichuan Province as the study area and quantified the habitat status and future climatic suitable distribution area of golden snub-nosed monkeys. The testing results of the MaxEnt model matched relatively well with the current actual distribution, proving the reliable applicability of the model. The protected areas guaranteed biodiversity conservation, but the conservation effect only sometimes met expectations (Rodrigues et al. 2004). We found some suitable habitats in Liangshan, but we have not found any traces of golden snub-nosed monkeys in the area during our field surveys. According to historical records, Liangshan was once the historical distribution area of golden snub-nosed monkeys. Through research, the reason for this phenomenon was that the suitable habitat in Liangshan was a separate and limited plot, geographically isolated from the golden snub-nosed monkey groups in other areas, which was probably the main reason for the disappearance of golden snub-nosed monkeys in Liangshan.

Our research identified climate change–induced translocations in the habitat distribution and ecological corridors of golden snub-nosed monkeys. By means of a long-term field survey and historical data, we have collected relatively comprehensive distribution occurrences of golden snub-nosed monkeys in Sichuan Province, which provided sufficient reliability for habitat prediction. MaxEnt model has been widely used in predicting species distribution because of its superiority in species distribution prediction (Elith et al. 2011; Phillips and Dudík 2008). Circuit theory is one of the most superior methods to simulate species dispersal and has achieved certain success in the practice of wildlife corridor design (Dai et al. 2019). Based on the circuit theory, we used the MaxEnt outputs to simulate the dispersal paths of golden snub-nosed monkeys in different climate and land-use change scenarios. Using the integrated results of the two ecological models was not only conducive to identifying the priority conservation areas, but also conducive to the formulation of protection measures for the trans-regional ecological corridors. A minor weakness of this research was the adoption of climatic data with relatively coarse resolution, which did not include reference to the fine-scale data that have an impact on the long-term survival of golden snub-nosed monkeys. In addition, our study was specific to primate species conservation and may not be directly extrapolated to other species. Nevertheless, the conservation of golden snub-nosed monkeys in this region still benefits the survival of different species in the same region. The following recommendations were proposed to reduce the impact of climate change on this species: (1) protecting current habitats and establishing a cross-border conservation system for protected areas where golden snub-nosed monkeys are distributed, to ensure habitat connectivity and stability; (2) keeping dispersal paths accessible to ensure that the species can spread to northern habitats; (3) monitoring the movement trend and habitat quality of each subpopulation for an extended period, including physical features and vegetation group trends; and (4) establishing artificial intervention conservation mechanisms for extreme weather disasters, such as food supplies. Species viability analysis provides theoretical guidance for the conservation and management of endangered species, but many other uncertainties influence the survival time and status of species. The prediction results can only be limited to the general trend of the survival prospects of endangered species under specific conservation measures or threat factors with uncertainty. In natural environments, species primarily exist as heterogeneous populations. It is not advisable to determine the survival status of a species based only on the viability of a single population surviving in a particular habitat. Therefore, when analyzing the species’ population viability, the results of long-term research and monitoring of species’ population dynamics can more realistically reflect the actual status of the species.

### Supplementary Information

Below is the link to the electronic supplementary material.

Supplementary file1 (XLSX 20 KB)

Supplementary file2 (DOC 34 KB)

Supplementary file3 (DOC 116 KB)

Supplementary file4 (DOC 64 KB)

## Data Availability

The original contributions presented in the study are included in the article, further inquiries can be directed to the corresponding author.
