# Supplementary material for: Climate change and anthropogenic activities shrink the range and dispersal of an endangered primate in Sichuan Province, China
Source: Environ Sci Pollut Res Int. 2023 Nov 18;30(58):122921–33. doi: 10.1007/s11356-023-31033-2 (PMC10724096; doi:10.1007/s11356-023-31033-2)
Supplement: Supplementary file 2 — Supplementary file2 (DOC 34 KB) [file 11356_2023_31033_MOESM2_ESM.doc]

Appendix 2 Environmental factor definitions.

| Code | Environmental factors | Unit |
| --- | --- | --- |
| Bio1 | Mean Annual Temperature | ℃ |
| Bio2 | Mean Diurnal Range | ℃ |
| Bio3 | Temperature Constancy | － |
| Bio4 | Temperature Seasonality (standard deviation *100) | － |
| Bio5 | Max Temperature of Warmest Month | ℃ |
| Bio6 | Min Temperature of Coldest Month | ℃ |
| Bio7 | Temperature Annual Range (Bio5-Bio6) | ℃ |
| Bio8 | Mean Temperature of Wettest Quarter | ℃ |
| Bio9 | Mean Temperature of Driest Quarter | ℃ |
| Bio10 | Mean Temperature of Warmest Quarter | ℃ |
| Bio11 | Mean Temperature of Coldest Quarter | ℃ |
| Bio12 | Annual Precipitation | mm |
| Bio13 | Precipitation of Wettest Month | mm |
| Bio14 | Precipitation of Driest Month | mm |
| Bio15 | Precipitation Seasonality (Coefficient of Variation) | － |
| Bio16 | Precipitation of Wettest Quarter | mm |
| Bio17 | Precipitation of Driest Quarter | mm |
| Bio18 | Precipitation of Warmest Quarter | mm |
| Bio19 | Precipitation of Coldest Quarter | mm |
| ELE | Elevation | m |
| HII | Human Influence Index | － |
| LUCC | Land-Use and Land-Cover Change | － |
