# Supplementary material for: Climate change and anthropogenic activities shrink the range and dispersal of an endangered primate in Sichuan Province, China
Source: Environ Sci Pollut Res Int. 2023 Nov 18;30(58):122921–33. doi: 10.1007/s11356-023-31033-2 (PMC10724096; doi:10.1007/s11356-023-31033-2)
Supplement: Supplementary file 3 — Supplementary file3 (DOC 116 KB) [file 11356_2023_31033_MOESM3_ESM.doc]

| **Appendix 3 Pearson correlation coefficient of environmental variables** | | | | | | | | | | | | | | | | | | | | | | |
| --- | --- | --- | --- | --- | --- | --- | --- | --- | --- | --- | --- | --- | --- | --- | --- | --- | --- | --- | --- | --- | --- | --- |
|  | **Bio1** | **Bio2** | **Bio3** | **Bio4** | **Bio5** | **Bio6** | **Bio7** | **Bio8** | **Bio9** | **Bio10** | **Bio11** | **Bio12** | **Bio13** | **Bio14** | **Bio15** | **Bio16** | **Bio17** | **Bio18** | **Bio19** | **ELE** | **HII** | **LUCC** |
| **Bio1** | 1.000 |  |  |  |  |  |  |  |  |  |  |  |  |  |  |  |  |  |  |  |  |  |
| **Bio2** | -0.871 | 1.000 |  |  |  |  |  |  |  |  |  |  |  |  |  |  |  |  |  |  |  |  |
| **Bio3** | -0.758 | 0.925 | 1.000 |  |  |  |  |  |  |  |  |  |  |  |  |  |  |  |  |  |  |  |
| **Bio4** | 0.273 | -0.432 | -0.728 | 1.000 |  |  |  |  |  |  |  |  |  |  |  |  |  |  |  |  |  |  |
| **Bio5** | 0.979 | -0.867 | -0.828 | 0.448 | 1.000 |  |  |  |  |  |  |  |  |  |  |  |  |  |  |  |  |  |
| **Bio6** | 0.992 | -0.910 | -0.775 | 0.243 | 0.960 | 1.000 |  |  |  |  |  |  |  |  |  |  |  |  |  |  |  |  |
| **Bio7** | -0.712 | 0.725 | 0.414 | 0.295 | -0.572 | -0.778 | 1.000 |  |  |  |  |  |  |  |  |  |  |  |  |  |  |  |
| **Bio8** | 0.993 | -0.888 | -0.812 | 0.370 | 0.991 | 0.983 | -0.657 | 1.000 |  |  |  |  |  |  |  |  |  |  |  |  |  |  |
| **Bio9** | 0.990 | -0.834 | -0.677 | 0.146 | 0.946 | 0.987 | -0.773 | 0.970 | 1.000 |  |  |  |  |  |  |  |  |  |  |  |  |  |
| **Bio10** | 0.990 | -0.895 | -0.829 | 0.402 | 0.995 | 0.980 | -0.640 | 0.997 | 0.963 | 1.000 |  |  |  |  |  |  |  |  |  |  |  |  |
| **Bio11** | 0.990 | -0.838 | -0.678 | 0.139 | 0.944 | 0.988 | -0.779 | 0.969 | 0.999 | 0.962 | 1.000 |  |  |  |  |  |  |  |  |  |  |  |
| **Bio12** | 0.700 | -0.800 | -0.680 | 0.174 | 0.665 | 0.741 | -0.682 | 0.686 | 0.685 | 0.698 | 0.699 | 1.000 |  |  |  |  |  |  |  |  |  |  |
| **Bio13** | 0.528 | -0.566 | -0.342 | -0.201 | 0.415 | 0.574 | -0.752 | 0.491 | 0.563 | 0.478 | 0.574 | 0.760 | 1.000 |  |  |  |  |  |  |  |  |  |
| **Bio14** | 0.683 | -0.810 | -0.818 | 0.489 | 0.720 | 0.710 | -0.467 | 0.703 | 0.633 | 0.723 | 0.636 | 0.786 | 0.333 | 1.000 |  |  |  |  |  |  |  |  |
| **Bio15** | -0.540 | 0.642 | 0.744 | -0.591 | -0.631 | -0.543 | 0.175 | -0.575 | -0.465 | -0.604 | -0.474 | -0.612 | 0.016 | -0.840 | 1.000 |  |  |  |  |  |  |  |
| **Bio16** | 0.546 | -0.597 | -0.375 | -0.183 | 0.437 | 0.594 | -0.763 | 0.507 | 0.577 | 0.498 | 0.590 | 0.829 | 0.983 | 0.404 | -0.083 | 1.000 |  |  |  |  |  |  |
| **Bio17** | 0.679 | -0.811 | -0.823 | 0.494 | 0.719 | 0.707 | -0.459 | 0.700 | 0.628 | 0.721 | 0.632 | 0.809 | 0.357 | 0.994 | -0.849 | 0.427 | 1.000 |  |  |  |  |  |
| **Bio18** | 0.487 | -0.530 | -0.289 | -0.284 | 0.364 | 0.539 | -0.765 | 0.441 | 0.531 | 0.427 | 0.543 | 0.782 | 0.977 | 0.358 | -0.013 | 0.986 | 0.380 | 1.000 |  |  |  |  |
| **Bio19** | 0.677 | -0.809 | -0.823 | 0.497 | 0.717 | 0.704 | -0.456 | 0.698 | 0.625 | 0.719 | 0.629 | 0.805 | 0.353 | 0.995 | -0.848 | 0.423 | 1.000 | 0.376 | 1.000 |  |  |  |
| **ELE** | -0.969 | 0.918 | 0.871 | -0.448 | -0.982 | -0.963 | 0.622 | -0.983 | -0.932 | -0.987 | -0.934 | -0.725 | -0.490 | -0.746 | 0.639 | -0.513 | -0.749 | -0.439 | -0.746 | 1.000 |  |  |
| **HII** | 0.738 | -0.694 | -0.644 | 0.283 | 0.733 | 0.732 | -0.503 | 0.744 | 0.715 | 0.743 | 0.719 | 0.562 | 0.441 | 0.547 | -0.429 | 0.452 | 0.545 | 0.412 | 0.542 | -0.746 | 1.000 |  |
| **LUCC** | -0.402 | 0.358 | 0.310 | -0.099 | -0.393 | -0.402 | 0.298 | -0.402 | -0.397 | -0.398 | -0.400 | -0.313 | -0.216 | -0.283 | 0.259 | -0.230 | -0.280 | -0.204 | -0.279 | 0.397 | -0.253 | 1.000 |
