# Supplementary figures and images for: Climate change and anthropogenic activities shrink the range and dispersal of an endangered primate in Sichuan Province, China
Source: Environ Sci Pollut Res Int. 2023 Nov 18;30(58):122921–33. doi: 10.1007/s11356-023-31033-2 (PMC10724096; doi:10.1007/s11356-023-31033-2)

**Appendix 4 Statistical graphs of MaxEnt model output results.**


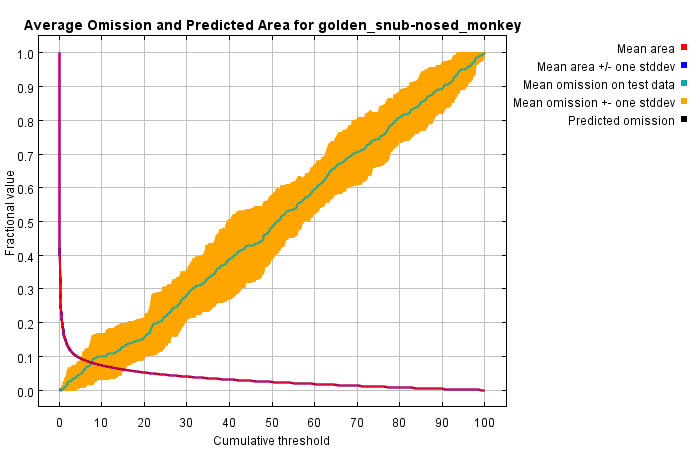


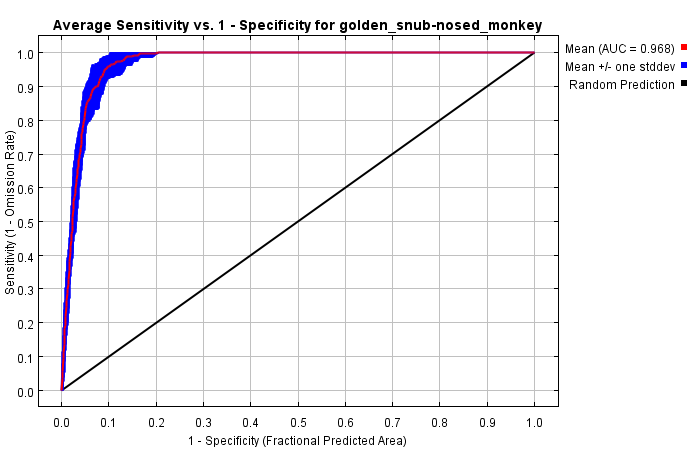

Supplement: Supplementary file 4 — Supplementary file4 (DOC 64 KB) [file 11356_2023_31033_MOESM4_ESM.doc]
